# Contextual and individual determinants of oral health-related quality of life among adolescents

**DOI:** 10.1590/1807-3107bor-2024.vol38.0019

**Published:** 2024-03-11

**Authors:** Ana Paula Milagres Alfenas SILVA, Jessica Klockner KNORST, Julia Rodrigues ANDRADE, Rafaela Silveira PINTO, Renata Castro MARTINS, Amália MORENO, Fabiana VARGAS-FERREIRA

**Affiliations:** (a) Universidade Federal de Minas Gerais – UFMG, School of Dentistry, Department of Community and Preventive Dentistry, Belo Horizonte, MG, Brazil.; (b) Universidade Federal de Santa Maria – UFSM, School of Dentistry, Department of Stomatology, Santa Maria, RS, Brazil.; (c) Universidade Federal de Minas Gerais – UFMG, School of Dentistry, Department of Community and Preventive Dentistry, Belo Horizonte, MG, Brazil.; (d) Universidade Federal de Minas Gerais – UFMG, School of Dentistry, Department of Dentistry, Belo Horizonte, MG, Brazil.

**Keywords:** Adolescent, Social Determinants of Health, Multilevel Analysis, Quality of Life

## Abstract

The aim of this study was to assess the factors associated with oral health-related quality of life in adolescents (OHRQoL). Individual data on adolescents were collected from a secondary database. OHRQoL was measured using the oral impact on daily performance (OIDP) scale. Individual- and city-level variables were selected to represent the structural and intermediate determinants of health. The individual covariates analyzed were sex, age, skin color, maternal education, household income, number of people per room in the housing unit, dental attendance, self-perception of dental needs, untreated dental caries, and gingival bleeding. The contextual variables included the allocation factor, the Human Development Index (HDI), Gini coefficient, illiteracy, unemployment, income, average number of emergency dental visits per inhabitant, access to a sanitary sewer system, garbage collection, primary health care coverage, oral health team coverage, and number of tooth extractions between selected dental procedures and supervised toothbrushing. Unadjusted and adjusted multilevel Poisson regression analyses were used to evaluate the relationship between contextual and individual variables with overall OIDP scores (STATA version 16.0) - rate ratio (RR) and 95%CI. The mean OIDP score was 0.72 and the prevalence was 31.8%. There was an association between supervised toothbrushing average and the outcome (RR 0.95; 95%CI 0.91–0.99). Moreover, adolescents who lived in municipalities with the highest average number of emergency dental visits per inhabitant showed a higher OIDP. Sex, maternal education, untreated dental caries, and gingival bleeding were associated with OIDP. In addition, intersectoral public policies focusing on the reduction of social inequalities should be on the agenda of policymakers and stakeholders.

## Introduction

Oral health-related quality of life (OHRQoL) indicators have been developed to assess people’s satisfaction and comfort in relation to their oral health whilst performing their activities of daily living.^1^ The association between demographic and socioeconomic factors, dental clinical status, psychosocial factors, and OHRQoL indicators has been investigated, mainly in adolescents.^2-4^ Thus, OHRQoL is an important outcome that results from the interaction between oral health conditions and general health, social, and contextual factors.^5^ Moreover, OHRQoL is an adjunct to clinical parameters in planning public health policies and in evaluating oral health strategies.

Adolescence is the stage of development between the ages of 10 and 19 years,^6^ during which individuals may undergo psychosocial changes and be exposed to risks.^7^ Providing individuals with healthcare at this stage can be challenging, considering the extensive transformation and maturation they go through.^8^ Therefore, understanding the patient can aid both clinical decision-making and evaluation of interventions, services, and programs, especially in populations in need of treatment, as oral health and regular dental care can be promoted in adulthood.^9^ In addition, improving social conditions through intersectoral actions should be on the political agenda to enhance adolescents’ oral health and quality of life.

Most studies with adolescents evaluate OHRQoL in terms of clinical aspects and/or socioeconomic issues addressed at the individual level. Generally, adolescents with a worse socioeconomic background at an individual level are more exposed to risk factors associated with oral health problems.^2-4^ However, this socioeconomic issue needs to be better addressed within a social context. In studies involving adolescents and adults, a direct correlation has been demonstrated between their social environment and OHRQoL.^10-11^ Health inequities are disparities in disease distribution among population groups that are considered unfair, unnecessary, and avoidable and that arise from social and economic differences.^12^ Social inequalities have been characterized by differences in the oral health of adolescents according to income level and education, which eventually undermines the access to and the utilization of oral healthcare.^13^ It is therefore essential to assess health outcomes using multilevel analysis, which allows making inferences at the contextual and individual levels,^14^ thus contributing to the better understanding of the population needs and to the formulation of more public policies.

Accordingly, the aim of the present study was to investigate the factors associated with OHRQoL in adolescents in a Brazilian state. The null hypothesis stated that the outcome would be associated with socioeconomic and clinical factors.

## Methodology

In 2012, the Minas Gerais Oral Health Study was performed to evaluate the oral health status of residents of the state of Minas Gerais, in southeastern Brazil.^15^ Minas Gerais is the second most densely populated and the third wealthiest state in Brazil according to gross domestic product (GDP) data.^15^ The state has 853 municipalities. The municipalities that participated in the study (n = 57) were grouped into three broad domains: State capital, Inland towns I, and Inland towns II, based on the “town/city allocation factor,” employed in distributing state tax revenue for healthcare.^16^ The Inland towns I group included more autonomous/less vulnerable municipalities, whereas the Inland towns II group consisted of less autonomous/more vulnerable municipalities. Prior to the study, the research protocol was approved by the Research Ethics Committee of the Pontificia Universidade Católica de Minas Gerais, Brazil.

The sample size was estimated based on the prevalence of the oral impact of daily performance (OIDP). The following parameters were analyzed: prevalence of OIDP (34.5%)^4^, 95% confidence interval (CI), and 80% power. The sample should include at least 348 adolescents. In addition, a design effect of 2.0 was applied, resulting in a sample of 996 adolescents. An additional 10% was added to account for loss to follow-up and refusal to participate, totaling 1,006 sampled individuals. Further information is provided in the literature.^17,18^

All examiners and assistants underwent training and calibration exercises prior to the study. Inter-examiner agreement (Cohen’s kappa) was > 0.65. The examinations were performed in a well-lit room with the aid of clinical mirrors and probes in compliance with the World Health Organization recommendations.^19^ Moreover, the decayed, missing and filled teeth (DMFT) index and the community periodontal index (CPI)], questionnaires addressing socioeconomic aspects, the utilization of dental services, and dental treatment needs were assessed by the same calibrated team.

For this study, data on adolescents aged 15 to 19 years were extracted from the database of the Minas Gerais Oral Health Study. OHRQoL measured by the OIDP scale was the dependent variable. This scale serves as a sociodental indicator designed to measure the impact of factors that prevent people from performing activities of daily living. It measures oral impacts by means of nine questions on physical aspects (eating and enjoying food, speaking and pronouncing words, and cleaning teeth), psychological aspects (sleeping and relaxing, smiling, laughing, displaying one’s teeth without embarrassment, and maintaining emotional well-being without becoming irritated), and social aspects (working,fulfilling a social role, and finding satisfaction in social gatherings and physical activity).^20^ The higher the absolute value, the greater the individual’s negative experience. The variables were considered at two levels: individual variables (Level 1) and contextual (local) variables (Level 2). Level 1 comprised sex, age, skin color, maternal education, household income, number of people per room in the housing unit, dental attendance, self-perception of dental needs, untreated dental caries, and gingival bleeding. Level 2 comprised allocation factor, the Human Development Index (HDI), Gini coefficient, illiteracy, unemployment, income, average number of emergency dental visits per inhabitant, access to a sanitary sewer system, garbage collection, primary health care coverage, oral health team coverage, and number of tooth extractions between selected dental procedures and supervised toothbrushing.^15,21^ All the variables are displayed in Table 1.

**Table 1:** Description of independent variables according to the level of analysis involving adolescents (n = 1,202), SB Minas Gerais, Brazil, 2012.

Variables	Description
Level 1 – Individual	
Sex	Male-female
Household income	Up to R$1,500 (Brazilian currency) (≤2BMW) *More than R$1,500 (>2 BMW)
Skin color	Self-reported skin color; a dichotomous variable was created from five original categories (white or non-white).
Age (years)	15–16 / 17–18
Maternal education (years)	Dichotomous: < 8 or ≥ 8
Number of people per room	Quantitative
Prevalence of untreated dental caries	Dichotomous: presence or absence
Gingival bleeding	Dichotomous: presence or absence
Self-perception of dental need	No need (healthy crown and root)
	One surface restoration
	Two or more surface restorations
	Prosthetic crown needed for any reason
	Dental facet
	Pulp treatment and restoration
	Tooth extraction
	White spot treatment
	Sealant
Dental attendance	Dichotomous: regular user or non-regular user
Level 2 – Local	
HDI	Human Development Index
Domain	State capital, Inland towns I, Inland towns II
Gini coefficient	Income or wealth distribution
Illiteracy	Percentage (%) of individuals who cannot read or write and have no language proficiency in the total resident population in the minimum age range in a geographic space within the considered year
Unemployment	Percentage (%) of economically active individuals unemployed during the reference week in a geographic space within the considered year
Half the BMMW	Percentage (%) of residents with monthly family income per capita up to half the Brazilian monthly minimum wage in a geographic space within the considered year
Oral health team coverage	Percentage (%) of population covered by Oral Health Teams
Primary health care coverage	Percentage (%) of the population covered by Primary Health Care teams
Supervised toothbrushing	Percentage (%) of collective actions of supervised toothbrushing
Rate of tooth extractions between selected dental procedures	Percentage (%) of extractions between all the dental procedures carried out
Sanitary sewage	Percentage (%) of residents with access to drinking water, sewage collection and treatment
Garbage collection	Percentage of residents with access to garbage collection system
Number of emergency dental visits per inhabitant	Average number of emergency dental visits per inhabitant in a given location and period

*$ Brazilian Real = $ 0.50 USD (Jul 2012); BMMW: Brazilian monthly minimum wage.

All the analyses were performed using the Complex Samples module to account for the complex sampling design of the of the Minas Gerais Oral Health Study.

Stata 16 (StataCorp.2014 Stata Statistical Software: Release 16.1. College Station, TX, StataCorp LP) was used for data analysis.

Unadjusted and adjusted multilevel Poisson regression analyses were used to evaluate the relationship between contextual and individual variables with overall OIDP scores. The multilevel model used the fixed-effects scheme with a random intercept. In this approach, we considered adolescents (level 1) nested into 57 municipalities (level 2). Our analysis was based on a theoretical framework developed by the Commission on Social Determinants of Health (Figure)^22^ and considered three models: Model 1 (“empty model”); Model 2 (“contextual”), which included contextual variables; and Model 3 (“full”), which comprised Model 2 and individual variables. This approach considered the hierarchy of variables (in which contextual variables influence individual variables). Variables with a p < .20 in the unadjusted analysis were considered for the adjusted models. In all models, the goodness of fit was measured using deviance (-2 log-likelihood) and the rate ratio (RR). The results were presented as RR and 95%CI.

**Figure:**
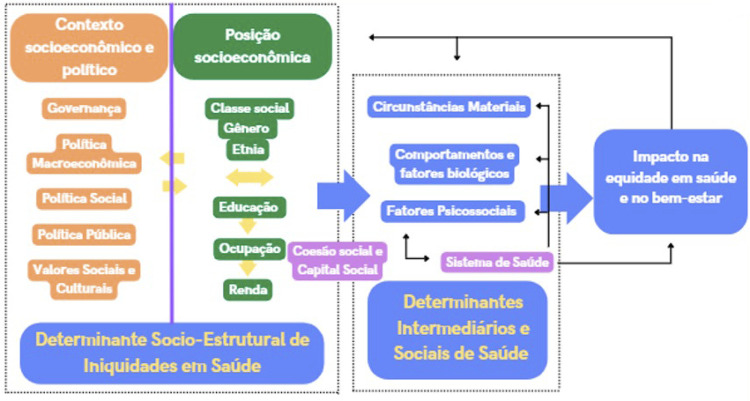
Conceptual model for assessing factors that affect the outcome.^22^

## Results

The study included 1,202 adolescents aged 15 to 19 years. Most of them were girls (55.3%) and non-white (59.6%) and had a lower household income (≤ 2BMW – 57.7%). The prevalence of untreated dental caries was 39.8%.

OIDP ranged from 0 to 9 points. The mean for oral impacts affecting activities of daily life (OIDP) over the past 6 months was 0.72 (SD 0.05), and the prevalence of oral impacts (OIDP ≥ 1) was 31.8% (95%CI 29.1–34.5). ‘Emotional well-being’, ‘cleaning teeth’, and ‘smiling’ were the most common aspects affected by oral health status. All the characteristics are shown in Table 2.

**Table 2:** Descriptive analysis of individual variables for the sample of adolescents (n = 1,202), Minas Gerais, Brazil, 2012.

Individual-level variables	n (%
Sex	
Male	533 (44.7)
Female	669 (55.3)
Age (years)	
15	294 (24.5)
16	252 (21.0)
17	224 (18.6)
18	248 (20.6)
19	184 (15.3)
Skin color	
White	469 (40.4)
Non-white	733 (59.6)
Maternal education	
> 8 years of formal education	941 (78.4)
≤ 8 years of formal education	260 (21.6)
Household income	
> 2 BMW	402 (42.3)
< 2 BMW	742 (57.7)

	mean (SD)

Household crowding	4.50 (1.59)
Dental attendance 8	n (%)*
Regular user	618 (51.6)
Non-regular user	578 (48.4)
Self-perception of dental needs	
No	619 (53.4)
Yes	583 (46.6)
Untreated dental caries*	
No	694 (60.2)
Yes	506 (39.8)
Gingival bleeding	
	814 (66.2)
	388 (33.8)

	mean (SD)

OIDP	
OIDP extent	0.72 (0.05)
	n (%)
Prevalence (OIDP ≥ 1)	382 (30.8)
Eating	89 (7.4)
Speaking	48 (4.0)
Cleaning teeth	133 (11.0)
Sleeping	89 (7.4)
Smiling	115 (9.6)
Emotional well-being	148 (12.1)
Social role	46 (3.8)
Social contact	68 (5.7)
Doing sports	3 (3.1)

*Missing values for some variables; **Sampling design taken into account.

Table 3 shows the unadjusted associations of contextual and individual variables and the extent of OIDP. Adolescents living in cities with a high average of emergency dental visits per inhabitant were associated with a greater extent of OIDP (RR = 1.08; 95%CI 1.02–1.16). In addition, sex, skin color, number of people per room in the housing unit, maternal education, and household income (> 2 BMW) were associated with the outcome. Adolescents with dental treatment needs were more likely to have the outcome (RR = 2.27; 95%CI 1.96–2.64). The mean extent of OIDP was significantly greater in adolescents with untreated dental caries (RR = 2.25; 95%CI 1.95–2.61) and with gingival bleeding (RR = 1.56; 95%CI 1.32–1.84).

**Table 3:** Unadjusted association between contextual and individual variables and overall OIDP scores, determined by multilevel Poisson regression .

Variables	RR (95%CI)	p-value
Contextual-level variables		
Gini coefficient	1.50 (0.01–112.96)	0.854
HDI	0.83 (0.02–24.13)	0.913
Unemployed	1.01 (0.94–1.10)	0.735
Illiteracy	0.99 (0.97–1.02)	0.906
Family income per person	0.99 (0.99–1.00)	0.793
Primary healthcare coverage	0.99 (0.98–1.00)	0.108
Oral health team coverage	0.99 (0.99–1.00)	0.378
Supervised toothbrushing	0.96 (0.91–1.00)	0.102
Gross Domestic Product	0.99 (0.99–1.00)	0.138
Allocation factor	0.59 (0.21–1.62)	0.305
Number of emergency dental visits per inhabitant	1.08 (1.02–1.16)	0.011
Sanitary sewer	0.99 (0.99–1.00)	0.711
Garbage collection	1.00 (0.99–1.02)	0.241
Number of tooth extractions between selected dental procedures	1.03 (0.99–1.07)	0.096
Individual-level variables		
Sex		< 0.001
Male	1.00	
Female	1.41 (1.22–1.63)	
Age (years)		0.329
15	1.00	
16	1.21 (0.99–1.49)	0.059
17	1.20 (0.98–1.49)	0.080
18	1.03 (0.83–1.28)	0.749
19	1.10 (0.87–1.39)	0.404
Skin color		< 0.001
White	1.00	
Non-white	1.36 (1.17–1.59)	
Maternal education		< 0.001
^3^ 8 years of formal education	1.00	
< 8 years of forma education	1.97 (1.66–2.35)	
Household income in R$		< 0.001
< 2 BMW	1.00	
> 2 BMW	0.57 (0.48–0.68)	
Number of people per room	1.11 (1.06–1.15)	< 0.001
Dental attendance		0.137
Regular user	1.00	
Non-regular user	1.11 (0.97–1.28)	
Self-perception of dental needs		< 0.001
No	1.00	
Yes	2.27 (1.96–2.64)	
Untreated dental caries		< 0.001
No	1.00	
Yes	2.25 (1.95–2.61)	
Gingival bleeding		< 0.001
No	1.00	
Yes	1.56 (1.32–1.84)	

RR: rate ratio; CI: confidence interval; BMW: Brazilian minimum wage (1BMW corresponds to approximately US$200).

The findings of the adjusted multilevel Poisson regression are illustrated in Table 4. Considering the contextual factors, adolescents who lived in cities with higher supervised toothbrushing showed a lower OIDP score (RR = 0.95; 95%CI 0.91–0.99). Moreover, individuals whose cities had a higher number of emergency dental visits per inhabitant presented a greater impact on quality of life (RR=1.08; 95%CI 1.01–1.14). Female adolescents (RR = 1.29; 95%CI 1.12–1.50) and those whose mothers had poor schooling (RR = 1.55; 95%CI 1.29–1.50) were more likely to present a higher OIDP score. The higher number of people per room in the housing unit, dental treatment needs, untreated dental caries (RR = 1.29; 95%CI 1.01–1.65), and gingival bleeding (RR = 1.25; 95%CI 1.06–1.49) influenced the OIDP score. Adolescents with a higher household income (> 2BW) were less likely to present a higher OIDP (RR = 0.66; 95%CI 0.55–0.79).

**Table 4:** Adjusted association between contextual and individual variables and overall OIDP scores, determined by multilevel Poisson regression

Variables	Model 1^a^	Model 2^b^	Model 3^c^

	“empty”	“contextual”	“full”

	RR (95%CI)	RR (95%CI)	RR (95%CI)
Fixed component			
Intercept	0.57 (0.46–0.70)	1.18 (0.58–2.39)	0.25 (0.13–0.45)
Contextual-level variables			
Gini coefficient	–	–	
HDI	–	–	
Unemployed	–	–	
Illiteracy	–	–	
Family income per person	–	–	
Primary healthcare coverage	–	–	
Oral health team coverage	–	–	
Supervised toothbrushing	–	0.95 (0.91–0.99)	0.95 (0.91–0.99)
Gross Domestic Product	–	0.99 (0.99–1.00)	0.99 (0.99–1.00)
Allocation factor	–		
Number of emergency dental visits per inhabitant	–	1.07 (1.01–1.13)	1.08 (1.01–1.14)
Sanitary sewer	–	–	
Garbage collection	–	–	
Number of tooth extractions between selected dental procedures	–	1.02 (0.98–1.05)	1.00 (0.97–1.05)
Individual-level variables			
Sex			
Male			1.00
Female			1.29 (1.12–1.50)
Age (years)			
15			
16			
17			
18			
19			
Skin color			
White			1.00
Non-white			1.17 (0.99–1.38)
Maternal education			
≥ 8 years of formal education			1.00
< 8 years of forma education			1.55 (1.29–1.87)
Household income in R$			
≤ 2 BMW			1.00
> 2 BMW			0.66 (0.55–0.79)
Number of people per room	–	–	1.06 (1.01–1.10)
Dental attendance	–	–	
Regular user			1.00
Non-regular user			0.99 (0.86–1.16)
Self-perception of dental needs			
No			1.00
Yes			1.83 (1.51–2.21)
Untreated dental caries			
No			1.00
Yes			1.29 (1.01–1.65)
Gingival bleeding			
No			1.00
Yes			1.25 (1.06–1.49)
Random component			
Deviance = (-2 log-likelihood)	31.176.876	3104.69	27.336.574
MRR	1.99	1.78	1.77
VPC	7.77	4.3	4.6

RR: rate ratio; CI, confidence interval; BMW, Brazilian minimum wage; VPC, variance partition coefficient; ^a^Model 1: empty model, representing an unconditional model. ^b^Model 2: mutually adjusted for contextual variables; ^c^Model 3: fully adjusted for contextual and individual variables. – variables not included and/or no associated with the outcome.

## Discussion

The present findings show that poor contextual variables and individual characteristics are related to poor OHRQoL. Adolescents living in municipalities with better dental services were less likely to have poor OHRQoL. In addition, living in households with more people per room, poor maternal education, low household income, untreated dental caries, gingival bleeding, dental treatment needs, and being female were associated with the outcome.

Adolescence is a period of gradual transition from childhood to adulthood, and it is characterized by physiological, psychological, and social changes.^23^ This phase is critical for health because adolescents are vulnerable to socioeconomic risk factors and, consequently, they are more likely to engage in unhealthy behaviors that may persist over time, including smoking and unhealthy oral hygiene practices.^24^ The literature has investigated OHRQoL among adolescents. OHRQoL is a multidimensional indicator that assesses the impact of oral diseases on daily functioning and on social, emotional, and psychological well-being of the individuals.^25^ Therefore, understanding the needs of the patient can aid both clinical decision-making and evaluation of interventions, services, and programs, especially in populations in need of treatment, such as oral health and regular care.^26^

In this study, female adolescents presented worse quality of life related to dental problems as compared to males, according to the literature.^26,27^ This is likely due to the fact that females report a higher prevalence of oral problems, have greater self-criticism about their dental appearance and also have low self-esteem.^26,28^ It is important to bear in mind that OHRQoL may be influenced by biological, cultural, and psychological factors, as well as expectations related to social roles.

In this study, low maternal education and higher household crowding had a negative impact on the adolescents’ OHRQoL. Furthermore, adolescents with a better household income showed better OHRQoL. Another interesting finding refers to household crowding. Generally, the influence of the poorer material circumstances on oral health can be explained by limited access to dental prevention and oral health promotion.

The effect of socioeconomic factors on OHRQoL inequalities can be explained by materialistic and psychosocial theories. Materialistic theories address the relationship between socioeconomic background and access to material resources, including health services.^29^ On the other hand, psychosocial theories focus on the perception and personal experience of living in unequal societies.^30^ It has been shown that household income may reflect the accumulation of knowledge, which influences the adoption of healthy habits and improves social conditions.^2^ Generally, individuals with a poor socioeconomic background are exposed to several risk factors that negatively impact oral health. However, adolescents with a high socioeconomic background tend to live in areas with higher health provision.^26^ All these aspects can contribute to and compromise their overall health, including oral health and OHRQoL.

Untreated dental caries is reported to be the main oral health outcome associated with a poorer quality of life among adolescents.^31^ Our finding is consistent with the literature.^31-33^ The literature has shown that children and adolescents with untreated caries seem to be more likely to experience pain and difficulty chewing foods and also report embarrassment when their anterior teeth are affected by caries.^33^ Untreated caries on permanent teeth is the most prevalent condition among all diseases, affecting 2.5 billion people worldwide.^34^ In addition, this finding suggests that more appropriate approaches and interventions are necessary to reduce and/or control dental caries.

Gingival bleeding was associated with the outcome. In this study, adolescents with gingival bleeding showed a higher OIDP score. Gingivitis is the most prevalent periodontal disease among children and adolescents.^33^ The literature has shown that socioeconomic determinants can induce gingival bleeding and that this condition can affect OHRQoL.^33^ For instance, the presence of dental calculus and gingivitis has psychosocial consequences, such as embarrassment when smiling and reluctance to brushing one’s teeth due to fear of gingival bleeding.^8^

Adolescents with dental treatment needs reported a higher OIDP. Dental treatment needs are important for the planning and implementation of oral health care services.^35^ Furthermore, such needs may be related to the utilization of dental services.

Two contextual variables were associated with the outcome. Recently, in Brazil, there has been an expansion of the program known as Family Health Strategy (FHS), and the FHS comprises a multidisciplinary team, such as oral health teams (OHTs).^36^ Adolescents who lived in municipalities with a larger availability of supervised toothbrushing exhibited a better OHRQoL. The literature has pointed out that the availability of a healthcare service could be translated into access, good-quality-services, and better OHRQoL, which has been proven to be a misconception in this population.^37^ Furthermore, adolescents who lived in municipalities with the highest average number of emergency dental visits per inhabitant showed a higher average OIDP. This finding is directly related to dental services offered to the population and to the presence of oral diseases. The literature has suggested that individuals who receive preventive dental care during their lifetime show a better OHRQoL.^38,39^ In addition, irregular dental visits have been reported to be associated with more untreated dental caries and poor OHRQoL.^38^ It is essential to improve oral health access and coverage in the municipalities, as well as to promote more equitable and regular dental services.

The present study has limitations that should be considered. The cross-sectional design does not allow determining the causal relationship between exposure and outcome. The main methodological strengths of this study are the use of a large representative population-based sample of Minas Gerais, providing better external validity. In addition, dental examiners were evaluated as highly reliable, providing adequate statistical power for the detection of important associations and multilevel analyses, which may contribute to a broader understanding of OHRQoL in adolescents. The statistical modeling was theoretically driven using an adapted theoretical framework on the social determinants of health proposed by the WHO, and it was very helpful in selecting the appropriate variables.^22^

The assessment of simultaneous contextual and individual indicators on adolescent’s OHRQoL can highlight the importance of the extent to which oral health problems are experienced by individuals living in different social contexts. In addition, an important clinical implication of the present study is that oral health promotion strategies aimed at reducing the prevalence of untreated dental caries and gingivitis have the potential to contribute to OHRQoL among adolescents.

From a health policy perspective, actions that promote comprehensive and interdisciplinary treatment of adolescents and understanding of the individual play an important role in improving the quality of life of adolescents.^26^ Finally, intersectoral public policies focusing on the reduction of social inequalities should be on the agenda of policymakers and stakeholders to enhance the OHRQoL and well-being of adolescents. Even though intersectoral policies have had some advances, they are not effective in practice. Brazil is a vast continental country, and this eventually leads to significant economic, social, and epidemiological inequalities. Intersectoral policies should be comprehensive and contextual, aimed at increasing access to health and oral services for both families and adolescents, thereby improving their quality of life.

## Conclusions

OHRQoL was influenced by contextual and individual variables. Adolescents who lived in municipalities with the highest average number of emergency dental visits per inhabitant showed a higher OIDP. Moreover, sex, maternal education, untreated dental caries, and gingival bleeding were associated with the outcome.
